# Acidic Urine Is Associated With Poor Prognosis of Upper Tract Urothelial Carcinoma

**DOI:** 10.3389/fonc.2021.817781

**Published:** 2022-01-24

**Authors:** Jang Hee Han, Seung-hwan Jeong, Hyeong Dong Yuk, Chang Wook Jeong, Cheol Kwak, Ja Hyeon Ku

**Affiliations:** ^1^ Department of Urology, Seoul National University Hospital, Seoul, South Korea; ^2^ Department of Urology, Seoul National University College of Medicine, Seoul, South Korea

**Keywords:** urine pH, acidic, upper tract urothelial carcinoma, recurrence, survival

## Abstract

**Purpose:**

To assess the prognostic role of acidic urine (low urine pH) in upper tract urothelial cancer (UTUC).

**Materials and Methods:**

We reviewed patients enrolled in Seoul National University Prospectively Enrolled Registry for Urothelial Cancer-Upper Tract Urothelial Cancer (SUPER-UC-UTUC) who underwent surgical resection from March 2016 to December 2020 in Seoul National University Hospital (SNUH). Patients with non-urothelial cancer or those who are in condition at end-stage renal disease were excluded. Acidic urine was defined as urine pH ≤ 5.5.

**Results:**

A total of 293 patients with a mean age of 70.7 ± 9.5 years were enrolled in this study. Pre-operative laboratory results showed a mean estimated glomerular filtration rate (eGFR) of 64.1 ± 19.2 mL/min/1.73m^2^ and a mean urine pH of 5.86 ± 0.66. Patients were subdivided into low (pH ≤ 5.5) and high (pH > 5.5) urine pH for comparison. As a result, all variables were comparable except for the T stage, which was significantly higher in the low urine pH group (p = 0.017). Cox regression analysis was performed to assess the clinical impact of acidic urine on patient survival. Multivariate Cox regression analysis revealed that tumor multifocality (HR 2.07, p = 0.015), higher T stage (HR 1.54, p = 0.036), lymphovascular invasion (HR 1.69, p = 0.033), eGFR < 60 mL/min per 1.73 m^2^ (HR 1.56, p = 0.017), and acidic urine (HR 1.63, p < 0.01) independently decreased disease-free survival (DFS), while multifocality (HR 9.50, p < 0.01), higher T stage (HR 9.51, p = 0.001) and acidic urine (HR 10.36, p = 0.004) independently reduced the overall survival (OS).

**Conclusions:**

Acidic urine is independently associated with reduced DFS and OS in UTUC. Acidic urine contributing to acidic environment may promote acquisition of agressive behavior of UTUC.

## Introduction

Urothelial cell carcinoma (UCC), which consists of upper tract urothelial carcinoma (UTUC) and bladder cancer, is the fourth most common tumors with a steady increase in incidence (1, 2). Although UTUC is relatively uncommon compared with bladder cancer (5–10% of UCC) (3), a three- to four-fold higher rate of muscle invasion at diagnosis (4) leads to poor prognosis of patients. In fact, the 5-year survival rate is < 50% for patients with T2-T3 and < 10% for patients with T4 UTUC, which are substantially lower than the average survival rates in other cancers (5). Therefore, studies focused on identification and stratification of risk factors *via* orthogonal approaches, including genetic analysis and molecular subtyping to enhance survival outcomes (3, 6–11).

Acidic tumor microenvironment is a hallmark of malignant tumor cells and is attributed to the high rate of glycolysis in tumor cells accompanied by hypoxia (12). Similar to other tumors, UCC also reprograms tumor metabolism toward Warburg-like aerobic glycolysis, resulting in lactic acid synthesis and acidic tumor microenvironment. While normal cells undergo cell death under acidic conditions, tumor cells adapt to chronic acidic conditions (13). This adaptation leads to acquisition of invasiveness, stemness, and chemoresistance, which are all correlated with tumor progression and poor overall survival (14–17).

Unlike other tumor, urothelial cancer has a distinct environment in that roughly half of them are exposed to urine on one side. Urothelium is in direct contract with urine, and is known to buffer acidic urine for reabsorption into the bloodstream (18). Thus, they are the primary barrier exposed to proton gradient both directly and indirectly. Previous studies elucidating the role of extracellular acidic media in intracellular signaling leading to aggressive phenotypes highlight the importance of extracellular liquid composition in cancer cells.

Thus, we hypothesized that acidic urine may aggravate the acidity of tumor environment, and thereby contribute to invasive and intractable characteristics, resulting in reduced disease-free survival (DFS), and overall survival (OS). In this context, we assessed the prognostic value of pre-operative acidic urine (low urine pH) in UTUC indicated for surgical resection.

## Materials and Methods

### Ethics Approval and Informed Consent

This study was approved by the Institutional Review Board (IRB) of Seoul National University Hospital (IRB no. 2110-210-1271). Informed consent was waived owing to the retrospective study design. The study was performed in accordance with applicable laws and regulations, good clinical practices, and ethical principles as described in the Declaration of Helsinki.

### Patient Population

We reviewed patients enrolled in Seoul National University Prospectively Enrolled Registry for Urothelial Cancer-Upper Tract Urothelial Cancer (SUPER-UC-UTUC) who underwent surgical resection from March 2016 to December 2020 at the Seoul National University Hospital (SNUH). During the period, 315 patients were registered in our database. To eliminate confounding factors, patients diagnosed with non-urothelial cancer or those with end-stage renal diseases were excluded. A total of 293 patients were eligible for the final analysis.

### Measurement and Definition of Acidic Urine

Urine pH was measured with a reagent test strip in which methyl red and bromothymol blue yielded different colors at different pH values (8). Using the test strip, the pH of the midstream urine was determined based on the color in 30 to 60 seconds with 0.5 intervals. Acidic urine was defined as urine pH equal or less than 5.5 using the dipstick test based on the previous definition by Bono et al., suggesting that the average urine pH was 6.0 (19).

### Collected Parameters

Patients’ demographic and clinico-pathological data including gender, age at surgery, accompanying comorbidities, pre-operative imaging findings, pre-operative laboratory findings (serum glucose, cholesterol, creatinine, estimated glomerular filtration rate, urine pH), pathology (size, multiplicity, histology, grade, stage, lymphovascular invasion, etc.), and disease status (recurrence-free survival, and OS) were collected.

### Statistical Analysis

Differences in clinical and pathological characteristics of patients with low pH (≤ 5.5) and high pH (> 5.5) of urine were compared using independent Student’s t-test. Univariate and multivariate Cox regression analyses was performed to assess the independent influence of possible risk factors on DFS and OS. All statistical analyses were performed using SPSS version 25 software (SPSS, version 25.0.0.2, IBM Corp., Armonk, NY). A P value <.05 was considered statistically significant, and all statistical tests were two-sided.

## Results

This study enrolled 293 patients with a mean age of 70.7 ± 9.5 years, including 205 (70.0%) males. The mean body-mass index (BMI) was 24.8 ± 3.2 kg/m^2^. More than 80% of patients manifested pathologically proven high grade UTUC, associated predominantly with the renal pelvis (48.5%). Based on pre-operative laboratory results, the mean creatinine level was 1.15 ± 0.38 mg/dL and the mean estimated GFR was 61.1 ± 19.2 mL/min/1.73m^2^. The mean urine pH was 5.86 ± 0.66. Approximately 20% of patients had a previous history of bladder cancer and the median follow-up period was 27 months (**Table 1**).

**Table 1:** Baseline characteristics.

Total (n)	293
Age (yrs) (mean ± SD)	70.7 ± 9.48
Sex (Male) (n,%)	205 (70.0)
BMI (kg/m^2^) (mean ± SD)	24.8 ± 3.24
HTN (n, %)	164 (56.0)
DM (n, %)	79 (27.0)
Laterality (R: L)	133:160 (54.6:45.4)
Tumor size (cm)	3.76 ± 3.04
Tumor location (n, %)	
Renal pelvis	142 (48.5)
Upper ureter	57 (19.5)
Mid ureter	62 (21.2)
Low ureter	78 (26.5)
Multiplicity (n, %)	24 (8.0)
Preoperative lab	
Creatinine (mg/dL)	1.15 ± 0.38
eGFR (mL/min/1.73 m^2^)	64.1 ± 19.2
Urine pH	5.86 ± 0.66
Previous bladder cancer history (n, %)	62 (21.2)
Median follow-up period (months)	27.0 (12.7-40.3)

BMI, body mass index; HTN, hypertension; DM, diabetes mellitus; eGFR, estimated glomerular filtration rate.

With regard to peri-operative findings, about 97% of patients underwent radical nephroureterectomy, while 3% of patients were treated with segmental ureterectomy. Patients with high pathological T stage (n = 184, 61.1%) outnumbered those with low T stage. Pathologically proven lymph node metastasis was detected in 13 cases (4.4%). Lymphovascular invasion was seen in 41 patients (14%), while carcinoma *in situ* (CIS) was found in 87 patients (29.7%) (**Table 2**).

**Table 2:** Perioperative characteristics.

Total (n)	293
Type of surgery (n, %)	
Nephroureterctomy with bladder cuff resection	285 (97.3)
Segmental ureterectomy	8 (2.7)
Surgical method (n, %)	
Open	161 (54.9)
Minimally-invasive surgery	132 (45.1)
Bladder cuff resection method (n, %)	
Open	221 (77.5)
Minimally-invasive surgery	64 (22.5)
Operative time (minutes)	159.96 ± 62.80
Estimated blood loss (mL)	281.74 ± 378.14
Venous invasion (n, %)	17 (5.8)
Lymphovascular invasion (n, %)	41 (14.0)
Perineural invasion (n, %)	22 (7.5)
Carcinoma *in situ* (n, %)	87 (29.7)
Grade (n, %)	
High grade	247 (84.3)
Low grade	40 (13.7)
Pathologic T stage (n, %)	
T1-2	184 (61.1)
T3-4	111 (36.9)
Pathologic N stage (n, %)	
N0	51 (16.9)
N1-2	13 (4.4)

Patients were divided into two subgroups according to their urine pH. Low urine pH (≤ 5.5) was found in 163, while high urine pH (> 5.5) was detected in 130. Mean age and BMI were statistically comparable between the two groups. Sex distribution and number of patients diagnosed with hypertension or diabetes mellitus were also comparable. Number of patients with smoking history were also comparable between two groups. Laboratory findings indicated statistically similar levels of fasting plasma glucose, total cholesterol (representative markers for metabolic syndrome), serum creatinine, and estimated glomerular filtration rate (eGFR) (representative markers for kidney function) between the two groups. Ipsilateral hydronephrosis and tumor grade also showed no statistically significant difference between the two groups (p = 0.696 and 0.956, respectively). Patients with low urine pH tended to have higher T stage (p = 0.017). The two groups comprised a comparable number of patients with lymphovascular invasion, perineural invasion, venous invasion, and carcinoma *in situ*. Metastasis or death occurred significantly more frequently in patients with low urine pH (**Table 3**).

**Table 3:** Comparison of low (≤ 5.5) and high (> 5.5) urine pH groups.

	Urine pH ≤ 5.5 (n = 163)	Urine pH > 5.5 (n = 130)	p-value
Age (yrs)	70.1 ± 9.3	71.5 ± 9.8	0.214
Sex (Male)	119 (73.0)	86 (66.2)	0.204
BMI (mean ± SD)	25.0 ± 3.15	24.5 ± 3.42	0.232
Smoking history (n, %)	83 (50.9)	66 (50.8)	0.980
HTN (n, %)	89 (54.6)	75 (57.7)	0.596
DM (n, %)	51 (31.3)	28 (21.5)	0.062
Fasting plasma glucose (mg/dL)	121.3 ± 50.8	113.4 ± 30.6	0.132
Total cholesterol (mg/dL)	178.6 ± 39.7	180.8 ± 35.1	0.736
Creatinine (mg/dL)	1.16 ± 0.40	1.13 ± 0.36	0.629
eGFR (mL/min/1.73m^2^)	64.0 ± 18.8	64.2 ± 19.6	0.951
Hydronephrosis (n, %)	74 (45.4)	62 (47.7)	0.696
Laterality (Right)	73 (44.8)	60 (46.2)	0.815
Tumor grade (n, %)			
Low grade	22 (13.8)	18 (14.1)	
High grade	137 (86.2)	110 (85.9)	0.956
Pathologic T stage (n, %)			
T1-2	92 (56.4)	91 (70.0)	
T3-4	71 (43.6)	39 (30.0)	0.017
Pathologic N stage (n, %)			
N0	27 (16.6)	24 (18.5)	
N1-2	8 (4.9)	5 (3.8)	0.807
Multiplicity (n, %)	14 (8.6)	10 (7.7)	0.821
Perineural invasion (n, %)	15 (9.2)	7 (5.4)	0.454
Carcinoma *in situ* (n, %)	46 (28.2)	41 (31.5)	0.818
Lymphovascular invasion (n, %)	25 (15.3)	16 (12.3)	0.738
Venous invasion (n, %)	9 (5.5)	8 (6.2)	0.956
Adjuvant chemotherapy (n,%)	35 (21.5)	20 (15.4)	0.185
Metastasis (n, %)	39 (23.9)	19 (14.6)	0.047
Death (n, %)	15 (9.2)	3 (2.3)	0.015

BMI, body mass index; HTN, hypertension; DM, diabetes mellitus; eGFR, estimated glomerular filtration rate.

To evaluate appropriate risk factors for tumor recurrence and patient death, univariate and multivariate Cox regression analysis was performed. Univariate Cox regression analysis revealed that higher T stage [HR 1.84 (95% confidence interval of 1.28-2.64), p = 0.001], lymphovascular invasion [HR 2.07 (1.33-3.2), p = 0.001], low eGFR (< 60 mL/min/1.73m^2^) [HR 1.59 (1.11-2.27), p = 0.012], and low urine pH (≤ 5.5) (acidic urine) [HR 1.74 (1.20-2.53), p = 0.004] were associated with decreased DFS. Tumor multifocality [HR 3.48 (1.14-10.57), p = 0.028], higher T stage [HR 11.51 (3.32-39.87), p < 0.001], concomitant CIS [HR 2.55 (1.00-6.48), p = 0.049], lymphovascular invasion [HR 3.45 (1.29-9.22), p = 0.013], and low urine pH (≤5.5) [HR 4.71 (1.36-16.31), p = 0.014] were associated with decreased OS (**Table 4**).

**Table 4:** Univariate Cox regression analysis of disease-free survival and overall survival.

Variables	HR	95% CI	p-value
Disease Free Survival			
Age (yrs)	1.02	1.00-1.04	0.061
Sex (Male)	1.11	0.75-1.65	0.601
Previous Bladder cancer	1.43	0.96-2.15	0.081
Tumor size (cm)	1.04	0.99-1.09	0.168
Multifocality	1.72	0.97-3.06	0.065
High grade	1.55	0.89-2.71	0.124
High T stage (T3-4)	1.84	1.28-2.64	0.001
Lymph node positive	0.79	0.27-2.27	0.656
Concomittant CIS	1.40	0.97-2.04	0.075
Lymphovascular invasion	2.07	1.33-3.22	0.001
eGFR < 60 (mL/min/1.73 m^2^)	1.59	1.11-2.27	0.012
Adjuvant chemotherapy	0.99	0.63-1.58	0.997
Urine pH ≤ 5.5	1.74	1.20-2.53	0.004
Overall Survival			
Age (yrs)	1.02	0.98-1.07	0.355
Sex (Male)	1.13	0.40-3.18	0.814
Previous Bladder cancer	2.21	0.80-6.09	0.125
Tumor size (cm)	0.96	0.79-1.16	0.656
Multifocality	3.48	1.14-10.57	0.028
High grade	3.46	0.46-26.04	0.228
High T stage (T3-4)	11.51	3.32-39.87	<0.01
Lymph node positive	0.84	0.10-6.74	0.865
Concomittant CIS	2.55	1.00-6.48	0.049
Lymphovascular invasion	3.45	1.29-9.22	0.013
eGFR < 60 (mL/min/1.73m^2^)	1.24	0.49-3.12	0.649
Adjuvant chemotherapy	3.03	1.17-7.83	0.022
Urine pH ≤ 5.5	4.71	1.36-16.3	0.014

HR, hazard ratio; CI, confidence interval; CIS, carcinoma in situ; eGFR, estimated glomerular filtration rate.

**Figure 1:**
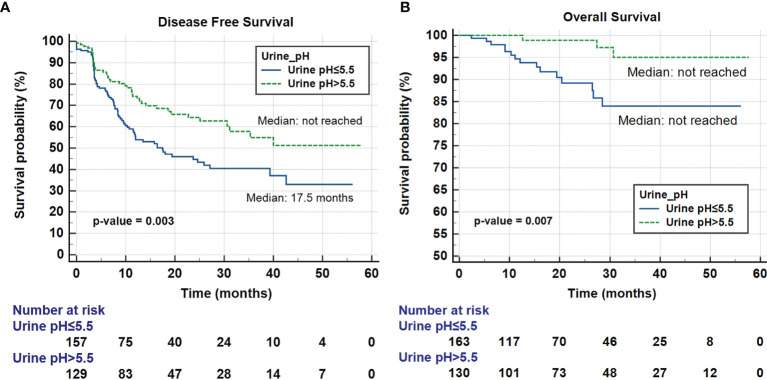
Kaplan-Meier curve of the effect of low urine pH (≤ 5.5) (blue) and high urine pH (> 5.5) (dotted green) groups on disease-free survival **(A)** and overall survival **(B)**.

Multivariate cox regression analysis revealed that tumor multifocality [HR 2.07 (1.15-3.72), p = 0.015], higher T stage [HR 1.54 (1.03-2.30), p = 0.036], lymphovascular invasion [HR 1.69 (1.04-2.75), p = 0.033], low eGFR (< 60 mL/min/1.73m^2^) [HR 1.56 (1.08-2.24), p = 0.017], and low urine pH (≤ 5.5) [HR 1.70 (1.16-2.49), p = 0.007] were independently associated with reduced DFS. Tumor multifocality [HR 9.50 (2.55-35.42), p = 0.001], higher T stage [HR 9.51 (2.47-36.55), p = 0.001], and low urine pH (≤ 5.5) [HR 10.36 (2.08-51.75), p = 0.004] were independent prognostic factors associated with OS (**Table 5**). **Figure 1** shows Kaplan Meier curve comparing DFS and OS between groups of patients with low and high urine pH.

**Table 5:** Multivariate Cox regression analysis of disease-free survival and overall survival.

Variables	HR	95% CI	p-value
Disease-Free Survival			
Multifocality	2.07	1.15-3.72	0.015
High T stage (T3-T4)	1.54	1.03-2.30	0.036
Lymphovascular invasion	1.69	1.04-2.75	0.033
eGFR < 60 (mL/min/1.73m^2^)	1.56	1.08-2.24	0.017
Urine pH ≤ 5.5	1.70	1.16-2.49	0.007
Overall Survival			
Multifocality	7.68	2.23-26.45	0.001
High T stage (T3-T4)	11.54	2.88-46.29	0.001
Concomitant CIS	1.54	0.58-4.09	0.387
Lymphovascular invasion	1.93	0.68-5.44	0.215
Adjuvant chemotherapy	0.72	0.25-2.08	0.542
Urine pH ≤ 5.5	5.83	1.53-22.29	0.010

HR, hazard ratio; CI, confidence interval; CIS, carcinoma in situ; eGFR, estimated glomerular filtration rate.

## Discussion

During the adaptation to acidic environment, various concurrent cellular events occur, such as increased levels of acid-induced cell motility, extracellular matrix degradation, and modified cellular and intercellular signaling, promoting invasion and metastasis (20, 21). Furthermore, extracellular acidosis also suppresses T cell-mediated immunity and attenuates immune responses (22). This phenomenon is an important aspect in immunotherapy. In fact, UTUC is currently under active investigation in many immunotherapy clinical trials, based on the results of studies investigating the other UCC, bladder cancer (23). The foregoing studies suggest that acidic tumor microenvironment is a clinical challenge and predicts failure of immunotherapy.

Many ongoing studies are actively developing drugs to neutralize the acidic environment using interfering pH-regulating systems, buffer therapy, or pH-sensitive drug-delivery system in various cancers, such as esophageal, colorectal, prostate, kidney, leukemia, and other solid tumors (13, 17). Based on the clinical and prognostic significance of acidic tumor microenvironment, we used clinical data to investigate whether acidic urine may further contribute to acidic environment (although not classical tumor microenvironment with extracellular matrix) resulting in shortened DFS and OS.

In the field of urology, urine pH is widely associated with urolithiasis (24); however, few studies focused on its relationship with urological cancer. In one study, Wright et al. reported that the relative risk for bladder cancer development increased in patients with acidic urine in a large population-based randomized clinical trial (25). In other recent study, Ide et al. reported that acidic urine (dipstick test based pH ≤ 5.5) is as independent predictor of upper tract recurrence in non-muscle-invasive bladder cancer patients with a smoking history (26). Consistent with the study by Ide et al., we found out acidic urine, which was defined as dipstick test based pH ≤ 5.5 is a significant prognostic factor. Furthermore, smoking group accompanying acidic urine was more vulnerable to UTUC disease recurrence compared to non-smoking group similar to the previous study (**Supplementary Figure 1** and **Supplementary Table 1**).

As urine pH is affected by multiple clinical factors, the independent effect of acidic urine on the prognosis of UTUC requires further investigation. First, the low urine pH was associated with metabolic syndrome in previous studies (27). Metabolic syndrome is known to be a significant risk factor for UTUC and consistently associated with tumor size and stage (28). Therefore, we evaluated factors associated with metabolic syndrome in patients with low and high urine pH. In our study, metabolic syndrome-related factors, such as BMI, history of diabetes mellitus, serum glucose and serum cholesterol were not significantly different between low and high urine pH groups. Second, as urine pH homeostasis is one of the primary functions of kidney, the acidity of urine may reflect the degree of renal function decline as demonstrated in previous studies (29). Because Cao-et al. reported that pre-operative renal insufficiency was a prognostic factor in UCC in a meta-analysis and systemic review, we further investigated the relationship between urine pH, renal function, and survival period. Consistent with a previous study, low eGFR was independently associated with shortened DFS in our study (**Table 4**). However, eGFR-based renal function was similar between patients with low and high urine pH. Furthermore, low eGFR and low urine pH were independent prognostic factors in multivariate Cox regression analysis. Accordingly, we concluded that urine pH is a powerful and independent prognostic factor in UTUC.

Meanwhile, the tumor stage tended to be higher in the group of patients with acidic urine (**Table 3**), suggesting that acidic urine may be involved in tumor progression, although further prospective studies are needed to elucidate the causal relationship. As reported previously (2, 7, 11, 30), tumor multifocality, lymphovascular invasion, and tumor stage were independently associated with a short DFS, and tumor stage was also significantly associated with OS. In addition to the well-known risk factors discussed above, acidic urine also independently reduced DFS and OS, further reinforcing our conclusion. With regard to surgical method, we performed nephroureterectomy by open or minimally invasive approach. Since there is conflicting evidence with regard to impact of surgical method on prognosis, we further analyzed its impact, and demonstrated there is no statistically significant survival difference between two surgical methods in our cohort (**Supplementary Figure 2**).

To summarize, in patients with acidic urine, low urinary pH may represent an extracellular acidic environment and trigger aggressive tumor cell growth and proliferation. Although many studies investigated the risk factors in UTUC, robust evidence was not available. Although a prospective study is still needed, our novel approach analyzing the role of urine pH may facilitate risk stratification of patients with UTUC.

The study included a well-designed prospective enrolled registry cohort with identical workflow from diagnosis to post-treatment follow-up. As urine pH measurement is a noninvasive technique, and can be repeated easily, it can be used even in the private clinic.

However, the study has several limitations. First, although this study has been performed using prospective database, we have relatively small sized cohort. To consolidate our result, large population based study should be followed. Second, only 21.8% of patients have undergone regional lymph node dissection (35 cases with hilar lymph node dissection, 26 cases with pelvic lymph node dissection) which could have led to inaccurate pathologic staging and could have affected oncologic outcome. Third, not every confounding factor affecting the acidity of urine, such as dietary intake or gastrointestinal ailments, such as vomiting or diarrhea has been considered. Fourth, measuring spot pH using dipstick test is associated with a methodological limitation in that 24-hour urine collection may reflect patient’s urine pH more accurately. Fifth, although urine pH represents a powerful prognostic factor in this study, prospective studies modulating urine pH and assessing its effect on survival period are needed to corroborate urine pH as an important risk factor. Furthermore, modulating extracellular pH and analyzing invasive metastatic features in *in vitro* and *in vivo* models will provide insight into the molecular mechanism of acidic urine associated with poor prognosis in UTUC.

## Conclusions

The study demonstrates the role of urine as a factor in the contributable environment of UTUC. Acidic urine is a significant prognostic factor in UTUC and facilitates patient risk stratification for appropriate treatment. Further studies will be conducted to investigate the molecular mechanism of acidic urine in aggressive tumors.

## Data Availability Statement

The original contributions presented in the study are included in the article/**Supplementary Material**. Further inquiries can be directed to the corresponding author.

## Ethics Statement

The studies involving human participants were reviewed and approved by Institutional Review Board (IRB) of Seoul National University Hospital. Written informed consent for participation was not required for this study in accordance with the national legislation and the institutional requirements.

## Author Contributions

JK had full access to all the study data and takes responsibility for the integrity of the data and accuracy of the data analysis. Study concept and design: JK. Acquisition, analysis, or interpretation of data: JH and JK. Drafting of the manuscript: JH and JK. Statistical analysis: JH, SJ, and HY. Administrative, technical, or material support: JH, CJ, CK, and JK. Study supervision: HY, CJ, CK, and JK. All authors contributed to the article and approved the submitted version.

## Conflict of Interest

The authors declare that the research was conducted in the absence of any commercial or financial relationships that could be construed as a potential conflict of interest.

## Publisher’s Note

All claims expressed in this article are solely those of the authors and do not necessarily represent those of their affiliated organizations, or those of the publisher, the editors and the reviewers. Any product that may be evaluated in this article, or claim that may be made by its manufacturer, is not guaranteed or endorsed by the publisher.

## Supplementary Material

Supplementary Figure 1 Kaplan-Meier curve of the effect of low urine pH (≤ 5.5) (blue) and high urine pH (> 5.5) (dotted green) groups on disease-free survival in patients without a smoking history **(A)** and patients with a smoking history **(B)**.

Supplementary Figure 2 Kaplan-Meier curve of the effect of open surgical approach (blue) and minimally invasive surgical approach (dotted red) on disease-free survival and overall survival.
