# Supplementary figures and images for: Acidic Urine Is Associated With Poor Prognosis of Upper Tract Urothelial Carcinoma
Source: Front Oncol. 2022 Jan 24;11:817781. doi: 10.3389/fonc.2021.817781 (PMC8818799; doi:10.3389/fonc.2021.817781)

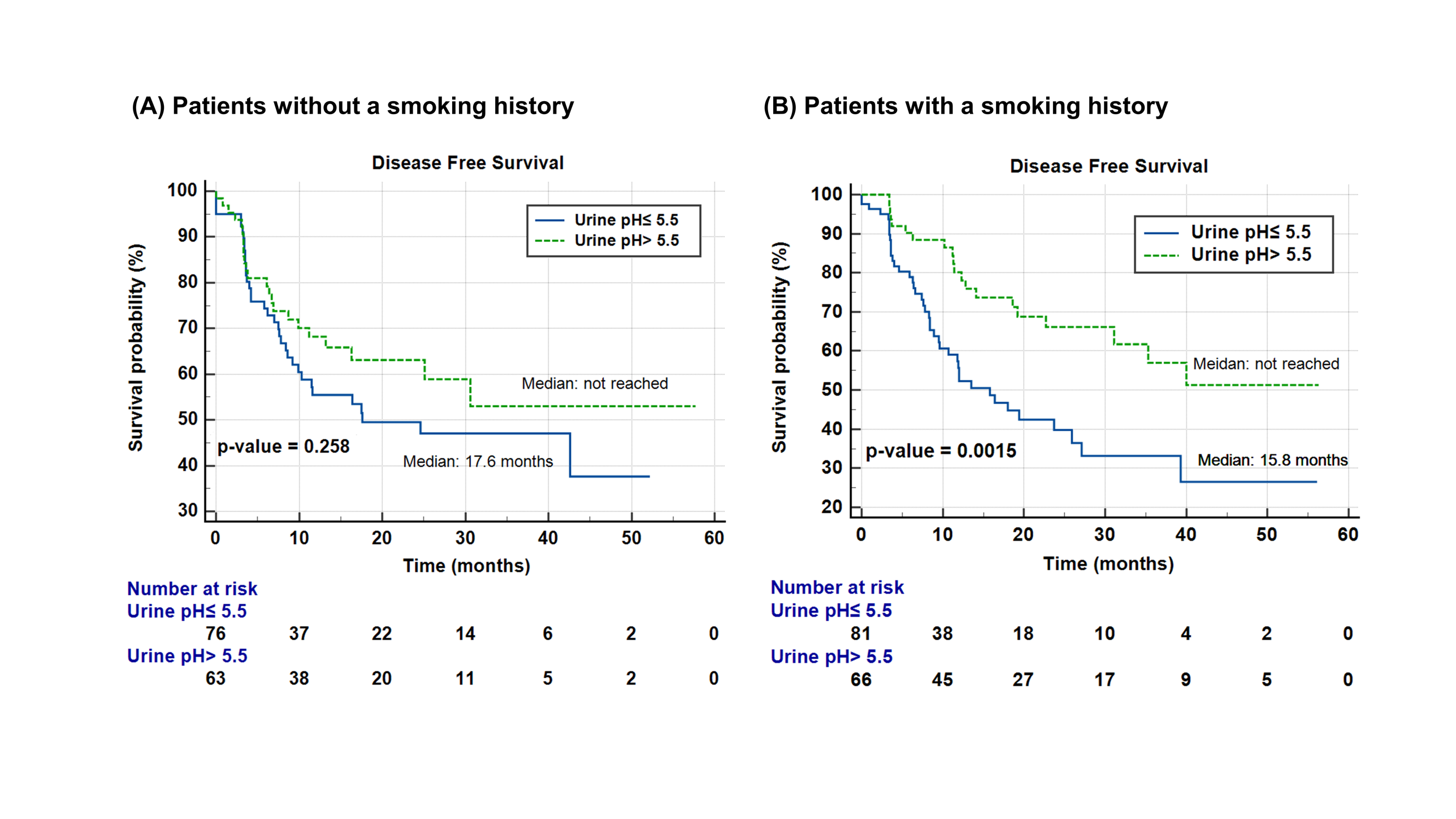

Supplement: Supplementary Figure 1 — Kaplan-Meier curve of the effect of low urine pH (≤ 5.5) (blue) and high urine pH (> 5.5) (dotted green) groups on disease-free survival in patients without a smoking history (A) and patients with a smoking history (B). [file Image_1.tif]

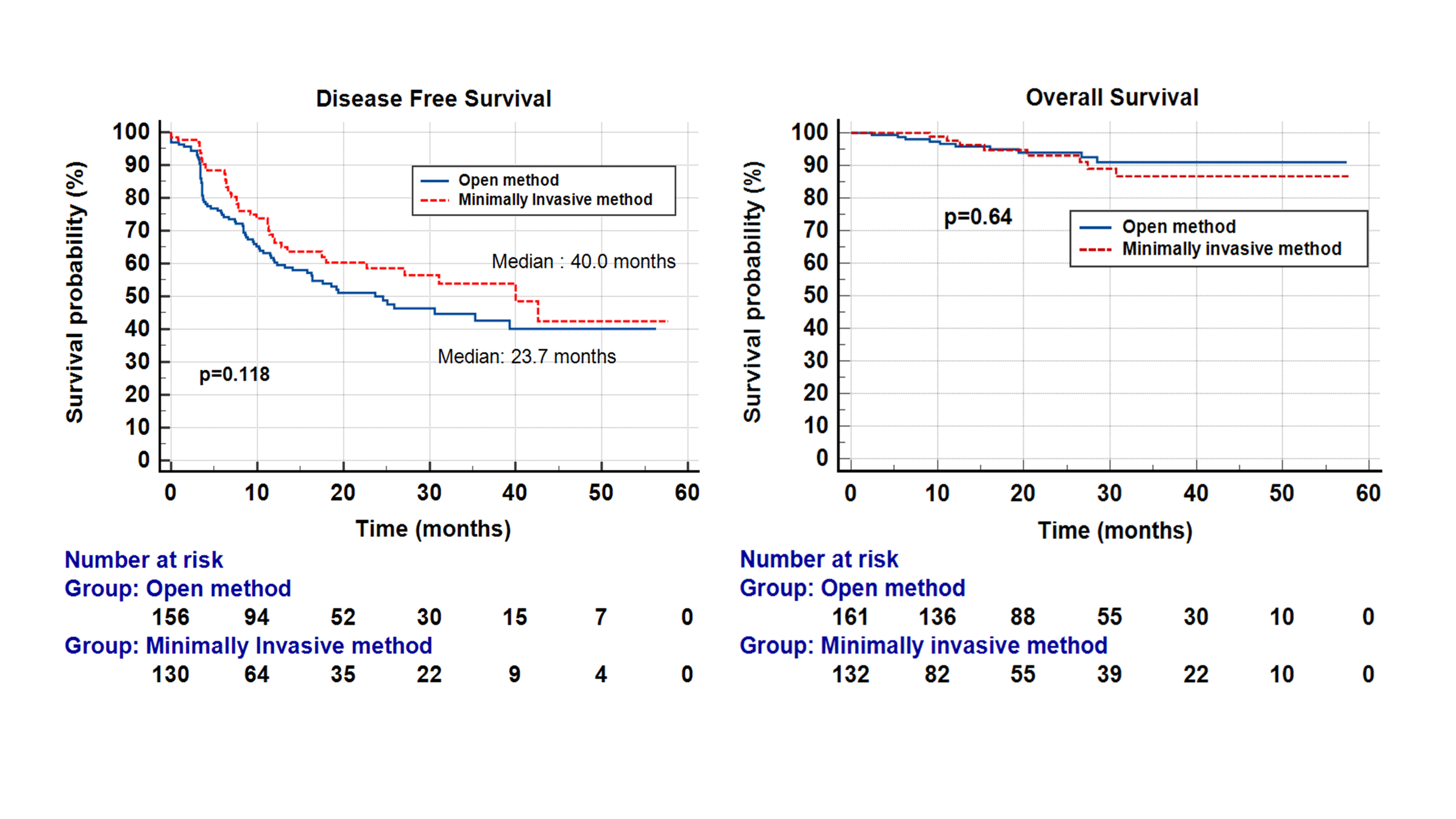

Supplement: Supplementary Figure 2 — Kaplan-Meier curve of the effect of open surgical approach (blue) and minimally invasive surgical approach (dotted red) on disease-free survival and overall survival. [file Image_2.tif]
