# Supplementary material for: Acidic Urine Is Associated With Poor Prognosis of Upper Tract Urothelial Carcinoma
Source: Front Oncol. 2022 Jan 24;11:817781. doi: 10.3389/fonc.2021.817781 (PMC8818799; doi:10.3389/fonc.2021.817781)
Supplement: Supplementary file 3 [file DataSheet_1.docx]

**Supplementary Table 1. Univariate Cox regression analysis of disease-free survival and overall survival in patients with or without smoking history**

|  | **Smoking history (-)** | | |  | **Smoking history (+)** | | |
| --- | --- | --- | --- | --- | --- | --- | --- |
|  | HR | 95% CI | p-value |  | HR | 95% CI | p-value |
| Disease Free Survival |  |  |  |  |  |  |  |
| Urine pH ≤ 5.5 | 1.35 | 0.80-2.28 | 0.261 |  | 2.32 | 1.36-3.97 | 0.002 |
|  |  |  |  |  |  |  |  |
| Overall Survival |  |  |  |  |  |  |  |
| Urine pH ≤ 5.5 | 3.79 | 0.82-17.57 | 0.088 |  | 6.06 | 0.73-50.60 | 0.096 |

HR: hazard ratio, CI: confidence interval
